# Diagnostic performance of digital breast tomosynthesis in female patients with nipple discharge

**DOI:** 10.1002/cnr2.1602

**Published:** 2022-02-10

**Authors:** Sanja Barsic Ostojic, Lora Grbanovic, Ana Tonklin, Lucija Kovacevic, Zlatko Marusic, Maja Prutki

**Affiliations:** ^1^ Clinical Department of Diagnostic and Interventional Radiology University Hospital Centre Zagreb Zagreb Croatia; ^2^ Department of Rheumathology and Rehabilitation General Hospital Dr. Tomislav Bardek Koprivnica Croatia; ^3^ Clinical Department of Pathology and Cytology University Hospital Centre Zagreb Zagreb Croatia; ^4^ School of Medicine University of Zagreb Zagreb Croatia

**Keywords:** breast cancer, digital breast tomosynthesis, nipple discharge

## Abstract

**Background:**

Nipple discharge is one of the most common symptoms related to the breast, but it is a presenting feature of breast cancer in 5%–12% of women.

**Aims:**

The purpose of this study was to determine the diagnostic performance of digital breast tomosynthesis (DBT) in the evaluation of patients with nipple discharge and to compare it with mammography (MMG), ultrasound (US), and magnetic resonance imaging (MRI).

**Methods and Results:**

This retrospective study included 53 patients with nipple discharge. All patients underwent DBT, and results were compared to MMG, breast US, and MRI. Radiological findings for each method were categorized according to BI‐RADS classification: categories 1–2 were considered negative and categories 3–5 positive. If a tissue specimen was obtained, the final diagnosis was established based on the results of histopathological analysis; otherwise, a clinical follow‐up was required for at least 2 years to confirm benign radiological findings. Measures of diagnostic accuracy of DBT, MMG, US, and MRI were calculated and compared.

**Results:**

Final histopathological analysis revealed six malignant breast lesions, all of which were detected in patients with pathologic nipple discharge. DBT and MRI exhibited high sensitivity (100%) and high negative predictive value (100%) for the detection of breast cancer in patients with nipple discharge. DBT showed higher specificity compared to MRI (82.9% vs. 61.9%). Sensitivity and specificity of MMG were 83.3% and 76.6%, respectively. Breast US was determined to have a sensitivity of 66.7% and specificity of 57.5%.

**Conclusion:**

DBT exhibited higher specificity than MRI at the same level of sensitivity and negative predictive value. Therefore, the use of DBT should be considered as an alternative to MRI in the assessment of patients with nipple discharge.

## 1 INTRODUCTION

Nipple discharge is the third most reported complaint related to the breast after breast pain and palpable breast mass.^1^ Up to 80% of women in their reproductive years will experience at least one episode of nipple discharge.^2^ Although majority of these cases are of benign origin, nipple discharge can be a source of anxiety for patients and can cause concern in physicians.^3^ It accounts for 2%–5% of medical visits among women, but most importantly, it is a presenting feature of breast cancer in 5%–12% of women.^4^

Clinically, nipple discharge can be categorized as physiologic or pathologic. Physiologic discharge is usually bilateral. It can be transparent or colored (but never contains blood. The most common causes of physiologic nipple discharge are pregnancy, lactation, nipple stimulation, endocrine abnormalities, and medications. Pathologic nipple discharge is usually unilateral. It can be bloody or serous. Although pathologic nipple discharge can indicate the presence of breast cancer, it can also be caused by intraductal papilloma, duct ectasia, or mastitis.^5^

Evaluation of non‐lactating patient with nipple discharge should begin with thorough history and physical examination. Cytologic analysis of nipple discharge is not routinely recommended in diagnostic workup of nipple discharge.^4^, ^6^ If initial evaluation suggests physiologic nipple discharge, imaging examination is not indicated. Additional imaging examination is required for patients with pathologic nipple discharge due to the associated increased risk of malignancy.

Initial diagnostic approach in the evaluation of patients with pathologic nipple discharge includes mammography (MMG) and ultrasound (US).^1^, ^6^ Magnetic resonance imaging (MRI) is appropriate for further evaluation of patients with negative MMG and US.^6^, ^7^

There are several studies evaluating digital breast tomosynthesis (DBT) galactography (ductography) for nipple discharge workup, comparing it with traditional 2D digital mammography ductography, but there were no reports published on the diagnostic performance of DBT in the evaluation of nipple discharge.^8^, ^9^

The aim of this study is to assess the value of DBT in evaluating patients with nipple discharge and to compare it with MMG, US, and MRI.

## 2 METHODS

This retrospective study was granted approval by the institutional review board and all data had been fully anonymized before they were accessed. All procedures performed in this case series involving human participants were in accordance with the ethical standards of the institutional and/or national research committee and with the 1964 Helsinki Declaration and its later amendments or comparable ethical standards.

**FIGURE 1:**
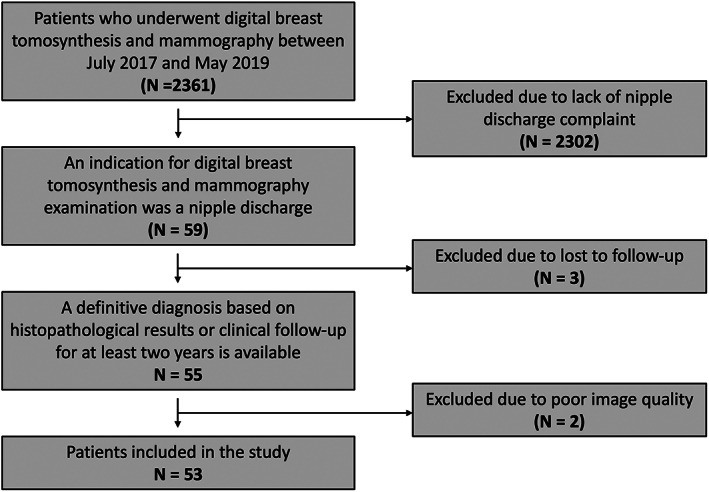
Flow chart showing selection of study participants

The electronic radiology information system was reviewed, and 2361 female patients underwent DBT and mammography between July 2017 and May 2019 were identified as shown in Figure 1. Patients who did not have nipple discharge (*N* = 2302), were lost to 2 years follow up (*N* = 3) and had poor quality DBT (*N* = 2) were not included in the study. Finally, 53 patients met the eligibility criteria and were included in the study.

DBT was performed on Selenia Dimensions (Hologic, Bedford, MA) unit in two standard views (craniocaudal and mediolateral oblique). The X‐ray tube rotated around the compressed breast within an angle range of 15° (−7.5° to +7.5°). Voltage ranged from 25 to 49 kV, max. 200 mA. Direct flat panel detectors made with amorphous selenium, 24 × 29 cm^2^, pixel size 140 μm were used. The filter was made of aluminum. Image acquisition was performed using continuous exposure method—rapid, short exposure pulses during continuous motion of the X‐ray tube with acquisition time of 5 s or less per breast. Reconstruction was performed after acquisition: slice thickness was 1 mm, time of reconstruction was 2–5 s and reconstructed pixel size was approximately 100 μm. C‐view software was used to generate 2D images from DBT. Those images were used as digital mammograms for the purpose of comparing diagnostic values of MMG and DBT.

Breast US was performed by breast imaging radiologists using a 11–15 MHz linear‐array transducer (LOGIQ S8, GE Healthcare Limited, Buckinghamshire, UK). Real‐time spatial compound imaging was used. The assessment started with evaluation of the axilla and the breast. The transducer was then positioned on the areola and angled beneath the nipple. Radial scan of the periareolar region was performed to delineate dilated lactiferous ducts and to detect intraductal tumors.

MR images were acquired on a 1.5 T scanner (Avanto, Siemens, Erlangen, Germany). Patients underwent imaging in the prone position with the breasts immobilized inside breast coil. Imaging protocol consisted of the following sequences: Short tau inversion recovery (STIR) sequence, axial T2‐weighted images without fat saturation and axial 3D fat‐saturated T1‐weighted images. One unenhanced image was obtained before, and five dynamic contrast‐enhanced images were obtained after contrast injection. Paramagnetic contrast material (Dotarem, Guerbet) was injected intravenously using automated contrast injector at a dosage of 0.1 mmol/kg and a rate of 3.5 ml/s, followed by a 15 ml saline flush.

Radiological findings for each imaging method were classified according to BI‐RADS classification: categories 3–5 were considered positive and categories 1–2 negative.^10^

Cytological analysis of nipple discharge was performed. Results were classified as positive if malignant cells were present, or negative if no malignant cells were found.

The final diagnosis of radiographically detected lesions was determined according to the results of histopathological analysis. In case the lesion was detected on breast US, tissue specimen was obtained using ultrasound‐guided core needle biopsy (CNB). If the lesion was visible on MMG, but could not be detected using US, a vacuum‐assisted stereotactic biopsy using mammographic guidance was performed. Local anesthetic was administered prior to the collection of tissue samples. Ultrasound‐guided CNB was performed using an automatic biopsy gun with 16‐G needle. Six 22‐mm‐long samples were taken from each lesion. In vacuum‐assisted stereotactic biopsy, 6–12 samples, each 15 or 22 mm long, were taken from lesions with 9‐G needle.

Patients with pathologic nipple discharge and negative radiological findings (BI‐RADS 1–2) underwent surgical duct excision based on intraoperative galactography with the purpose of making a histopathological diagnosis. Intraoperative galactography was performed under general anesthesia. The duct secreting the suspicious discharge was dilated with a probe and injected with methylene blue dye, which stained all segmental ducts that coalesce into marked main lactiferous duct. Glandular tissue was accessed through periareolar incision. The whole stained segment was excised and sent to histopathological analysis.

If a patient had a combination of physiologic nipple discharge and negative radiological findings, a clinical follow‐up was required for at least 2 years to confirm benign radiological findings. The follow‐up was performed at six‐monthly intervals.

Statistical analyses were performed with software packages in programming languages R 4.1.1 (exact2x2 1.6.5), as well as Python 3.8.5. (statsmodels 0.12.2, scipy 1.7.1, pandas 1.3.4). Sensitivity, specificity, positive predictive value (PPV), and negative predictive value (NPV) for detection of malignant disease were calculated for DBT, MMG, US, and MRI. The Exact McNemar test was used to assess the statistical significance of the observed differences in sensitivities and specificities between imaging modalities (MMG, US, MR imaging, and DTD). A p value of less than 0.05 was considered statistically significant.

## 3 RESULTS

The mean age of the patients was 54 years (range 31–86 years). Among 53 patients, 39 (73.6%) presented with pathologic nipple discharge and 14 (26.4%) with physiologic nipple discharge.

All 53 patients underwent DBT. A total of 14 (26.4%) positive (BI‐RADS categories 3–5) and 39 (73.6%) negative (BI‐RADS 1 and 2 category) radiological findings were identified using DBT. Calcifications were the most common finding on DBT in positive lesions, associated with 50% of malignant and 87.5% of benign lesions, followed by architectural distortion, mass and asymmetry. The mean diameter of calcification area was larger in malignant lesions (5.1 cm) than benign lesions (1.4 cm).

Synthesized 2D (s2D) images reconstructed from DBT were used as digital mammograms. A total of 16 (30.2%) positive and 37 (69.8%) negative radiological findings were detected using this technique. Calcifications were the most common finding in positive lesions on MMG as well.

Among negative lesions, mass was the most common finding on both MMG and DBT (47.8% and 52%, respectively), followed by dense breast tissue and architectural distortion. The least frequent findings in non‐suspicious lesions were calcifications, detected by both methods, and asymmetry, found only on DBT.

Breast US was performed on all patients, which revealed 24 (45.3%) positive and 29 (54.7%) negative radiological findings. Dilated ducts were the most common finding in positive lesions, accounting for 50% of findings in malignant lesions and 30% of findings in benign lesions. The second most frequent findings on US were cysts and masses. The mean duct diameter was marginally larger in malignant lesions and the mean mass diameter was larger in benign lesions. Cysts were the most frequent sonographic finding in negative lesions, followed by dilated ducts. Two out of six malignant lesions could not be detected on US and one malignant lesion falsely showed benign characteristics on US. Masses and heterogeneous area were the least common sonographic findings associated with negative lesions.

A total of 27 (50.9%) patients underwent MRI. A total of 14 (51.9%) positive and 13 (48.1%) negative radiological findings were identified using MRI. Non‐mass enhancement was the most common finding in positive lesions on MRI, while also being more prevalent in malignant lesions – 83.3% of malignant lesions and 50% of benign lesions presented as non‐mass enhancement on MRI. The most frequent finding on MRI in negative lesions was mass enhancement, followed by background enhancement, non‐mass enhancement and cysts.

Imaging characteristics of radiologically detected suspicious lesions are given in Table 1.

**TABLE 1:** Imaging characteristics of radiographically detected suspicious lesions

		Malignant lesions		Benign lesions	
Imaging method	Radiological findings	*n* (%)	Mean diameter (cm)	*n* (%)	Mean diameter (cm)
Mammography	Mass			3 (27.3)	0.7
	Asymmetry	1 (20)	2.4		
	Architectural distortion	1 (20)	1.0	2 (18.2)	1.2
	Calcifications	3 (60)	5.1	6 (54.5)	1.5
Digital breast tomosynthesis	Mass			1 (12.5)	1.1
	Asymmetry	1 (16.7)	2.4		
	Architectural distortion	2 (33.3)	2.2		
	Calcifications	3 (50)	5.1	7 (87.5)	1.4
Ultrasound	Mass	1 (25)	0.8	60 (30)	1.4
	Heterogeneous area	1 (25)	2.3	1 (5)	2.3
	Dilated duct	2 (50)	0.6	6 (30)	0.4
	Cyst			7 (35)	0.9
Magnetic resonance imaging	Mass	1 (16.7)	1.7	4 (50)	0.8
	Non‐mass enhancement	5 (83.3)	4.9	4 (50)	2

Cytologic examination of nipple discharge was performed in 38 (71.7%) patients, out of which 27 (71.7%) patients presented with pathologic nipple discharge and 11 (28.9%) patients had physiologic nipple discharge. The analysis identified 34 (89.5%) benign findings and four (10.5%) malignant findings. All patients with cytologically verified malignant findings presented with pathologic nipple discharge. Sensitivity of cytologic analysis for detecting malignancy was 75.0% and specificity was 79.4%.

Histopathologic evaluation was performed in 29 (54.7%) patients which either had a suspicious radiological finding or a combination of pathologic nipple discharge and negative radiological finding. Six (20.7%) malignant lesions and 23 (79.3%) benign lesions were revealed. All malignant lesions were identified in patients with pathologic nipple discharge. Histopathologic analysis identified 11.3% (6/53) malignant lesions among all patients with nipple discharge and 15.4% (6/39) malignant lesions in patients with pathologic nipple discharge. The most common malignant lesion detected in patients with pathologic nipple discharge was ductal carcinoma in situ – DCIS (50.0%), followed by invasive ductal carcinoma (33.3%) and Paget's disease of the breast (16.7%) (Table 2).

**TABLE 2:** Pathologic characteristics of lesions

Lesion type		*n*	%
Benign (fibroadenoma, fibrocystic changes, intraductal papilloma)		23	79.30
Malignant	DCIS	3	10.35
	Invasive ductal carcinoma	2	6.90
	Paget's disease of the breast	1	3.45
Total		29	100

Sensitivity, specificity, PPV, and NPV were calculated for each imaging method (Table 3). DBT and MRI exhibited the highest sensitivity and NPV (100%). While PPV of both methods was 42.9%, specificity of DBT was higher compared to MRI (83.0% vs. 61.9%). Mammography showed high sensitivity (83.3%) and specificity (76.6%) as well. PPV of MMG was relatively low (31.4%) and NPV was 97.3%. Of all radiological techniques, US exhibited the lowest sensitivity (66.7%), specificity (57.5%) and PPV (16.7%). NPV of US was 93.1%.

**TABLE 3:** Measures of diagnostic accuracy

	Sensitivity/%	Specificity/%	PPV/%	NPV/%
Digital breast tomosynthesis	100	83	42.9	100
Mammography (s2D)	83.3	76.6	31.4	97.3
Ultrasound	66.7	57.5	16.7	93.1
Magnetic resonance imaging	100	61.9	42.9	100

Exact McNemar test revealed significant difference in specificity between DBT and US (*p =* .02). We did not find a statistically significant difference in specificity between DBT and other imaging methods (MMG, MRI). We did not find a statistically significant difference in sensitivity between DBT and any other imaging method (MMG, US, MRI).

## 4 DISCUSSION

Nipple discharge is the presenting symptom of breast cancer in 5%–15% of patients.^2^, ^11^ American College of Radiology recommends DBT as one of the initial imaging methods for evaluation of patients with pathologic nipple discharge alongside MMG and US.6 Brandt et al. suggested that DBT could replace conventional MMG for evaluation of noncalcified lesions.^12^ In this study DBT exhibited higher sensitivity (100%), specificity (82.98%), PPV (42.86%) and NPV (100%) in comparison with MMG and US. Sensitivity, PPV and NPV of DBT were comparable to MRI. However, the only statistically significant difference was observed in specificity between DBT and US (*p =* .02). DBT showed the highest specificity of all imaging methods. These results are due to the ability of DBT to reduce masking effect of overlapping fibroglandular tissue, consequently improving detection of breast cancer.^13^

MMG is often performed as the initial imaging method in the evaluation of pathologic nipple discharge. However, sensitivity of MMG for detecting underlying malignancy in patients with pathologic nipple discharge can be relatively low due to the characteristics of some malignant lesions that can cause discharge, such as very small lesions, lesions without calcifications and completely intraductal lesions.^14^ Sensitivity of MMG in different reports ranges between 10.0% and 68.4%.^5^ In this study sensitivity of MMG was 83.3%. Higher sensitivity might be a result of using newer, more precise equipment. Variability of characteristics of malignant lesions in different studies could also have contributed to different sensitivity values.

US is suitable for examination of younger and/or pregnant patients, as well as other patients with mammographically dense breasts.^6^, ^15^ In this study sensitivity of US was 66.7% and specificity was 57.5%. In different studies sensitivity and specificity for detecting breast cancer in patients with pathologic nipple discharge range from 15.0% to 100.0% and from 31.0% to 99.6%, respectively.^5^, ^16^ Such variability could be explained by multiple factors that include differences in the criteria used to define pathologic nipple discharge and physiologic nipple discharge, variations in sonographic assessment of the breast and continuous improvement of US technology.^16^ In evaluation of pathologic nipple discharge, US can identify 63%–69% of lesions that are not visible on mammograms, therefore it is useful to combine US with MMG to achieve greater accuracy of diagnosis.^6^ In the publication of Yoon et al., sensitivity of combined MMG and US for detecting breast cancer in patients with pathologic nipple discharge was 82.4%, compared with 67.9% for MMG alone.^17^

Interestingly, in this study US correctly identified six negative lesions that DBT detected as positive. Philpotts et al. reported similar results in the context of breast cancer screening.^18^

Contrast‐enhanced MRI is the most sensitive imaging method for detecting breast pathology.^19^ Breast MRI should be considered when other radiological methods have failed to determine the underlying cause of pathological discharge from the nipple.^6^ It has high sensitivity not only for detecting malignant lesions (in situ or invasive), but also for detecting papillary lesions which often cause nipple discharge.^14^ Sensitivity of breast MRI for detecting the cause of pathologic nipple discharge ranges from 86% to 100% for invasive cancers and from 40% to 100% for noninvasive diseases.^6^ In this study, sensitivity was 100%, specificity 61.9%, PPV 42.7% and NPV 100%, similar to previously reported researches.^14^, ^20^ In this study MRI showed the same level of sensitivity and NPV, but lower specificity compared to DBT. These results suggest DBT as a suitable alternative imaging method to MRI in evaluation of patients with nipple discharge.

In this study all patients with an underlying malignancy presented with pathologic nipple discharge as reported in previous study.^16^ The incidence of breast cancer (in situ or invasive) among patients with pathologic nipple discharge was 15.4%. These results are in accordance with previous studies, in which reported incidence ranged from 5% to 23%.^5^, ^6^, ^21^ The most common malignant lesion detected in patients with pathologic nipple discharge was DCIS as it was reported in a previous study.^22^

In this study sensitivity and specificity of cytology were 75.0% and 79.4%, respectively. In different studies sensitivity varies between 11.1% and 74.5% and specificity varies from 30.0% to 99.5%.^23^, ^24^ Cytologic analysis is considered inappropriate as the only modality for evaluation of nipple discharge due to its variability in sensitivity and specificity and therefore should always be supplemented with imaging methods.^6^, ^23^, ^24^

There are several limitations to this study, most obvious being its retrospective design. Lack of statistical significance in sensitivity between DBT and other imaging method (MMG, US, MRI) may be due to a relatively sample size. Considering these limitations, prospective studies with larger sample size are warranted to establish accuracy of DBT in patients with nipple discharge.

## 5 CONCLUSION

DBT is a valuable addition to the evaluation of patients with nipple discharge. In the case of a positive DBT finding, US has value as a method to rule out the malignancy. Since DBT exhibited greater specificity than MRI at the same level of sensitivity and negative predictive value, it should be considered as an alternative to MRI in diagnostic workup of patients with nipple discharge.

## CONFLICT OF INTEREST

The authors declare no conflicts of interest.

## AUTHOR CONTRIBUTION

Sanja Barsic Ostojic: Data curation, conceptualization, methodology. Lora Grbanovic: Formal analysis: investigation. Ana Tonklin: Data curation, writing‐original draft. Lucija Kovacevic: Conceptualization, formal analysis, data curation, methodology. Zlatko Marusic: Formal analysis, investigation, methodology. Maja Prutki: Conceptualization, writing review and editing, methodology, formal analysis.

## ETHICS STATEMENT

The study received ethical approval by the Ethics committee of Clinical Hospital Centre Zagreb and School of Medicine University of Zagreb and was conducted according to the good clinical practice guideline, as well as the Declaration of Helsinki. All the patients signed informed consent and all data had been fully anonymized before they were accessed.

## Data Availability

The date that support the findings of this study are available from the corresponding author, Maja Prutki upon reasonable request.
